# Electron beam-formed ferromagnetic defects on MoS_2_ surface along 1 T phase transition

**DOI:** 10.1038/srep38730

**Published:** 2016-12-15

**Authors:** Sang Wook Han, Youngsin Park, Young Hun Hwang, Soyoung Jekal, Manil Kang, Wang G. Lee, Woochul Yang, Gun-Do Lee, Soon Cheol Hong

**Affiliations:** 1 Department of Physics and Energy Harvest-Storage Research Center (EHSRC), University of Ulsan, Ulsan 44610, Korea; 2 School of Natural Science, Ulsan National Institute of Science and Technology (UNIST), Ulsan 44919, Korea; 3 School of Materials Science and Engineering, Ulsan National Institute of Science and Technology (UNIST), Ulsan 44919, Korea; 4 Division of Physics and Semiconductor Science, Dongguk University, Seoul 04620, Korea; 5 Department of Materials Science and Engineering, Seoul National University, Seoul 08826, Korea

## Abstract

1 T phase incorporation into 2H-MoS_2_ *via* an optimal electron irradiation leads to induce a weak ferromagnetic state at room temperature, together with the improved transport property. In addition to the 1T-like defects, the electron irradiation on the cleaved MoS_2_ surface forms the concentric circle-type defects that are caused by the 2 H/1 T phase transition and the vacancies of the nearby S atoms of the Mo atoms. The electron irradiation-reduced bandgap is promising in vanishing the Schottky barrier to attaining spintronics device. The simple method to control and improve the magnetic and electrical properties on the MoS_2_ surface provides suitable ways for the low-dimensional device applications.

Recently, based on the reduced dimensionality of transition metal dichalcogenides (TMDs), the successful realization of field-effect transistors^1^ and the thickness-dependent, indirect-direct bandgap transition^2^,^3^ have boosted the development of two-dimensional (2D) materials for high-performance flexible electronic and optoelectronic devices^4^,^5^. Additionally, similar to graphene^6^, developing approaches to effectively induce the ferromagnetism into the diamagnetic 2H-MoS_2_ have attracted a great interest for possible spintronics and quantum information devices, but it is still challenging^7^,^8^,^9^,^10^,^11^,^12^,^13^,^14^,^15^,^16^,^17^,^18^,^19^,^20^,^21^,^22^. Extensive experimental and theoretical studies have effectively modified magnetic properties of MoS_2_ with the various forms of thin films^7^, nanoribbons^8^, nanosheets^9^, and even in bulk limit^10^,^11^. The used methods are by the formation of vacancies^11^,^12^,^13^, adsorbing non-metal atoms^14^,^15^ substitutional doping TM atoms^16^,^17^ and application of an external electric field^18^,^19^ or elastic strain^20^,^21^, and so on. Notably, together with high-Curie-temperature, relatively large ferromagnetism has been only realized in the significant presence of zigzag edges^7^,^22^. However, the possible degradation of transport properties due to the scattered morphology needs to be resolved for nanoscale device applications^23^.

The local introduction of the metallic 1 T phase into the 2 H matrix *via* Li intercalation^24^,^25^ or electron irradiation^26^ has been shown to improve the transistor performance^27^. Especially, novel fabrication methods using electron beam-based techniques have produced semiconducting MoS_2_ nanoribbons^28^ and metallic MoS_2_ nanowires^29^. Accordingly, for a magnetic MoS_2_, 1 T phase incorporation has been also explored by using the chemical exfoliation^30^,^31^,^32^. As a separate method, irradiation with low-energy electrons has been also used to modify the magnetic and transport properties^13^. However, although the achieved magnetic moments are remarkable^13^,^32^, it is still obscure whether the magnetism originates intrinsically from the existence of 1 T phase or edge effect driven by the various defects^20^,^33^. Thus, with a full understanding of 1 T phase incorporation, it is desirable to find more effective ways to control and improve the magnetic and electrical properties of MoS_2_. In this paper, we propose a simple method to improve transport property and induce room-temperature ferromagnetism through the optimal electron irradiation on the MoS_2_ surface. The magnetic moments are found to be attributed to the unpaired spins of Mo^4+^ ions induced by exotic defects, which form a specific shape of concentric circles on the surface region along the 2 H/1 T phase transition.

## Results and Discussion

**Figure 1:**
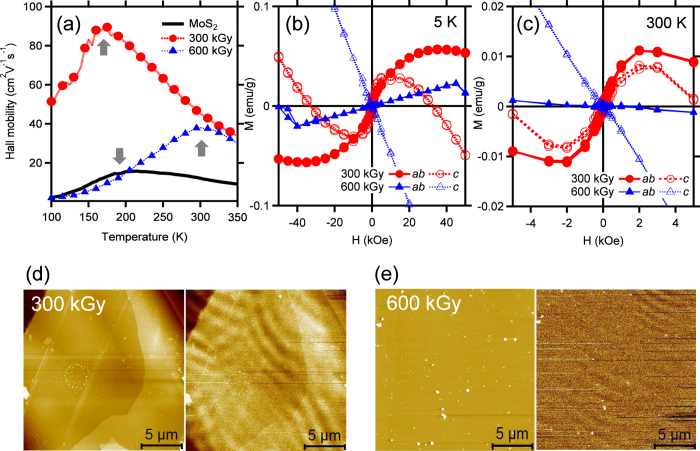
Comparison of single-crystalline MoS_2_ and electron-irradiated samples for (**a**) Hall mobility as a function of temperature, magnetic hysteresis loops of (**b**) 5 K and (**c**) 300 K, and (**d,e**) images of AFM (left) and MFM (right) with scan areas of 20 × 20 μm^2^. The magnetic field (H) is applied parallel (*ab*) and perpendicular (*c*) to the basal plane of samples.

We found a certain condition to increase and induce the Hall mobility and the diamagnetic to ferromagnetic phase transition, after electron irradiation on the cleaved MoS_2_ surfaces by changing the electron dose and the acceleration energy, respectively: the electron dose of 300 kGy (6.70 × 10^14^ electrons/cm^2^) and the acceleration energy of 0.7 MeV^34^,^35^. As shown in the temperature dependence of the Hall mobility (Fig. 1a), the electron irradiation of this condition improves the Hall mobility of the pristine MoS_2_, but slightly reduces a crossover temperature (T_C_, as indicated by arrow) of the pristine MoS_2_ (200 K) to 175 K. Above and below T_C_, the mobility is mainly subject to the phonon and impurity scatterings, respectively^36^,^37^. On the other hand, the higher electron dose of 600 kGy (1.39 × 10^15^ electrons/cm^2^) increases the T_C_ over room temperature. Such shift of T_C_ implies that the mobility is limited dominantly by charged impurities, while the phonon scattering plays a minor role.

Figures 1b shows the magnetizations as a function of the magnetic field strength (*H*) up to ±50 kOe at low (5 K) temperature. In comparison with the diamagnetic susceptibility^11^ of the pristine MoS_2_, the electron dose of 300 kGy induces the diamagnetic to a ferromagnetic phase transition. Interestingly, along the out-of-plane (the *c*-axis) direction, the diamagnetic behavior still remains for higher magnetic fields than ±10 kOe. The saturated magnetizations along the in-plane (the *ab*-plane) and out-of-plane directions are 0.057 emu/g (1.634 × 10^−3^ μ_B_/Mo ion) and 0.030 emu/g (8.60 × 10^−4^ μ_B_/Mo ion) at the *H* = 35 kOe and 1 kOe, respectively. These weak ferromagnetic states persist up to room temperature, but the saturated magnetizations of 5 K (Fig. 1b) are significantly reduced to 0.011 emu/g (0.315 × 10^−3^ μ_B_/Mo ion) and 0.008 emu/g (0.229 × 10^−3^ μ_B_/Mo ion) at the *H* = 2 kOe along the in-plane and out-of-plane directions, respectively (Fig. 1c). The coercivities (0.2 kOe) of both directions at 5 K are also reduced to 0.1 kOe at room temperature.

On the other hand, the higher electron dose of 600 kGy induces the diamagnetic to a paramagnetic phase transition along the in-plane direction while the out-of-plane direction still remains diamagnetic (Fig. 1b). Especially along the in-plane direction, the diamagnetic state also retains over the magnetic field of ±40 kOe, similarly to the case of the out-of-plane direction for the sample irradiated at 300 kGy. At room temperature, however, the temperature-dependent paramagnetic state disappears, while the relatively temperature-insensitive diamagnetic state remains (Fig. 1c)^10^,^11^. It is evident from the time-of-flight secondary ion mass spectroscopy measurements (not shown) that the electron irradiation of the current condition^34^,^35^ has influences on a few top layers of the cleaved MoS_2_ single crystals. Furthermore, the different magnetic states due to the different electron doses are elucidated in Fig. 1(d,e) of the atomic and magnetic force microscopy (AFM and MFM) images taken at room temperature. Similarly to shown in the previous study^11^, undulating magnetic domains representing the ferromagnetic state are clearly observed in the MFM image of 300 kGy (Fig. 1d), whereas the magnetic domains get much weakened in that of 600 kGy (Fig. 1e). This confirms that the electron dose of 300 kGy efficiently induces the ferromagnetic state on the MoS_2_ surface.

**Figure 2:**
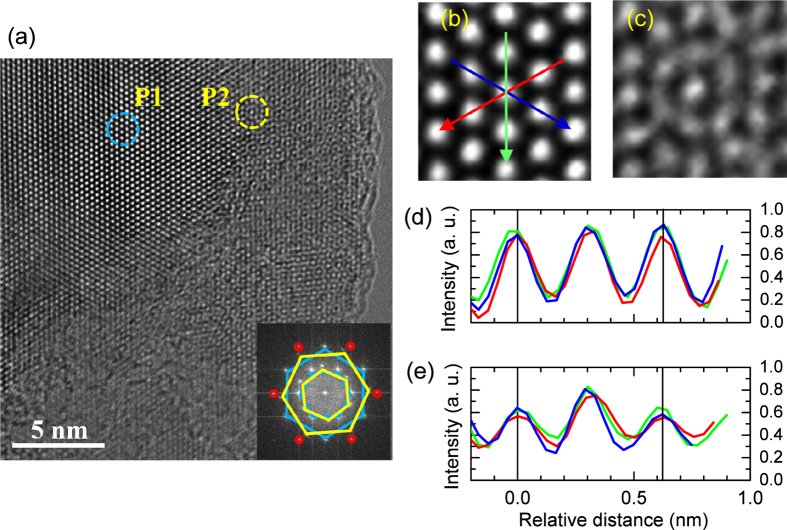
(**a**) HRTEM image of the electron-irradiated sample of 300 kGy. Two types of defects (P1 and P2) are magnified in (**b**) and (**c**), respectively. (**d,e**) Corresponding line profiles are obtained as the colored arrows in (**b**) and provide the estimated lattice constants of (**b**) *a* = 3.15 Å and (**c**) 3.11 Å, respectively. In the inset of (**a**), FFT image displays twisted (yellow and cyan) hexagons at the (100) and (110) planes. The six red circles indicate the (200) plane.

**Figure 3:**
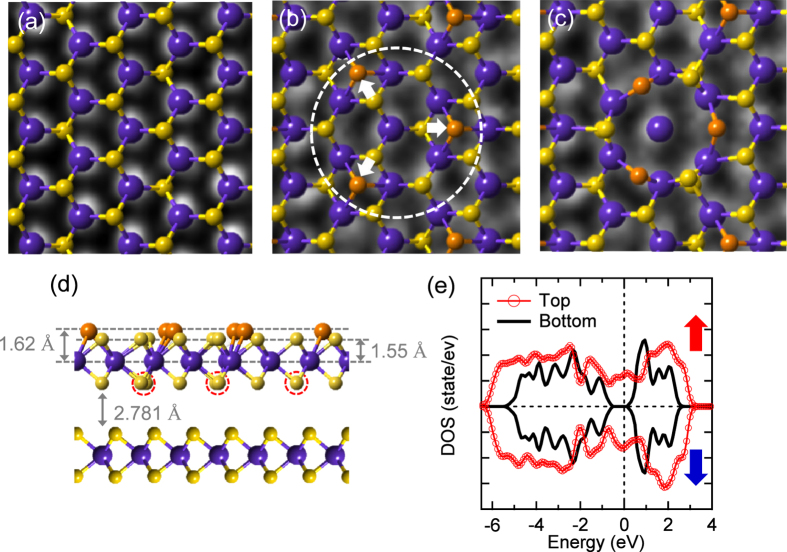
(**a**) Calculated 1 H phase of 2 × 2 supercells is overlapped in the TEM image of Fig. 2b. TEM image of Fig. 2c is compared to the calculated (**b**) 1T-like and (**c**) 1T-3V_S_ phases of 6 × 6 supercells. (**d**) Side view of 2L-MoS_2_. 1T-3V_S_ defect is formed by gliding the topmost (orange) S atoms and removing the bottom (dotted) S atoms at the top layer of 6×6 supercells of 2 H phase. Large (blue) and small (yellow and orange) spheres correspond to the Mo and S atoms, respectively. (**e**) Corresponding calculated total spin polarized DOSs of upper and bottom layers. Up and down arrows indicate positive and negative spin states. DOSs consist of the distribution of Mo 4*d* and S 3*p* electrons at the upper and bottom layers with a closed and ~0.64 eV bandgaps, respectively. The Fermi level is at zero energy.

To elucidate the magnetic domains in more detail, atomic structures on the electron-irradiated surface of 300 kGy were investigated by high-resolution transmission electron microscopy (HRTEM) after the sample was exfoliated using ultrasonic. The fringes of the HRTEM image (Fig. 2a) indicate that the thickness of the MoS_2_ layers is about mono- or bi-layers^38^. With the lack of honeycomb lattices, two types of defects are dominantly found; 1T-phase-like defect^24^,^25^,^26^ (P1) and concentric circle-type vacancies (P2). The inset of Fig. 2a shows the fast Fourier transform (FFT) image, where the inner and outer hexagons correspond to the (100) and (110) planes^39^,^40^. The two defects lead to having two (yellow and cyan) hexagons with a twist angle 24° at each plane, respectively. The electron irradiation-induced 1T-phase-like defect (Fig. 2b) is in good agreement with a previous study^26^ and supported by the negligible intensities between the main peaks of the line profiles^24^ (Fig. 2d). Additionally, the structural difference of the 1T-phase-like defect is confirmed by comparing of the TEM image with the 1 H phase (Fig. 3a), which is half of the unit cell of bulk 2H-MoS_2_. It is notable that the total energy of 1T-MoS_2_ is much higher than that of 1H-MoS_2_ by 0.8 eV^41^. However, the 1 H to 1 T phase transition can be driven by lowering the energy barrier *via* the charge injection of electron irradiation^42^.

**Figure 4:**
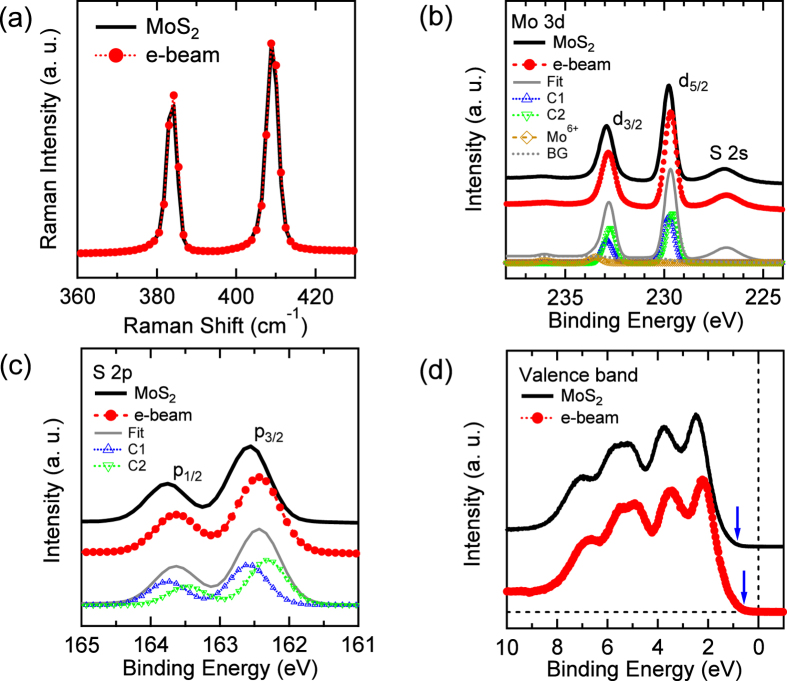
Comparison of (**a**) Raman and (**b**–**d**) XPS spectra between the single-crystalline MoS_2_ and the electron-irradiated sample (300 kGy), where Mo 3d and S 2p core-level spectra include the curve-fitting results of two components, C1 and C2, relating to the contribution of 2 H phase and electron irradiation-induced defects, respectively. BG indicates the background.

Now, we focus on the concentric-circle-type defect of Fig. 2c. Compared to the line profiles of Fig. 2d, the profiles of Fig. 2e indicate that the central and nearby atoms within the circle (Fig. 2c) correspond to the Mo and S atoms, respectively. In the (dotted) circle of Fig. 3b, the upper three (orange) S atoms of the 1 H phase glide as indicated by the arrows and form partly the 1 T phase. Then such the 1 T phase (Fig. 3b) is turned further into the concentric-circle-type phase (Fig. 3c) after pushing away the inner three S atoms denoted as dotted circles in Fig. 3d. Thus, we will refer to the latter phase as a 1T-3V_S_ defect from the vacancies of the inner three S atoms. The estimated lattice constant of the 1T-phase-like defect (Fig. 2d, *a* = 3.15 Å) is slightly reduced in the 1T-3V_S_ defect (Fig. 2e, *a* = 3.11 Å). The details are compared to the calculated results as described in the methods. However, the Raman signals of the characteristic 1 T phase^43^ observed in the chemically exfoliated MoS_2_ are not found in the electron-irradiated sample because of the finite thickness of the defect depth. Figure 4a shows two strong Raman peaks at 383 and 408 cm^−1^, which correspond to the E^1^_2g_ and A_1g_ modes, respectively. The Raman spectrum of the electron-irradiated sample is nearly identical to that of the pristine MoS_2_.

On the other hand, the calculations reveal that the 1T-3V_S_ defect doped bilayer MoS_2_ in a ferromagnetic state is more stable by energy difference of 0.420 eV per formula unit (fu) than a nonmagnetic one and has a magnetic moment of 0.084 μ_B_/Mo. Notably, the magnetic moment of the 1T-3V_S_ defect doped monolayer is 0.168 μ_B_/Mo. The thickness-dependent magnetic moment manifests that, compared to the calculated magnetic moments, the significantly reduced magnetic moments of the electron-irradiated samples (Fig. 1b,c) are attributed to the diamagnetic states of the subsurface layers remaining in the non-defective status. Furthermore, compared to the 1T-3V_S_ doped monolayer, the 1T-phase doped monolayer MoS_2_ (Fig. 3b) is more favored by the difference of 0.328 eV per fu and has a larger magnetic moment of 0.175 μ_B_/Mo (0.0875 μ_B_/Mo for bilayer MoS_2_). However, it is notable that 1T-3V_S_ defects were only obtained at the specific condition of 300 kGy, while the higher electron dose of 600 kGy mainly produced the 1T-phase-like defects (not shown) and induced the diamagnetic to the paramagnetic phase transition instead of the ferromagnetic phase. It is contrary to a simple consideration that the higher electron dose may produce more 1T-3V_S_ defects than 1T-phase-like defects. Thus, the 1T-phase-like defects are considered to be closely related to the V_S2_-like defects^33^, where each Mo atom lacks six nearby S atoms (3V_S2_). In other words, the remained S atoms of 1T-3V_S_ defects in Fig. 3c may be pushed away or moved further into the vdW gap at the higher electron dose. The first-principles calculations of 3V_S2_ (not shown) indicate that, similar to the V_S2_-doped monolayer MoS_2_, the 1 × 1 bilayer MoS_2_ is nonmagnetic (after removing the topmost S layer)^20^,^21^,^22^, and the 2 × 2 bilayer MoS_2_ is more likely to be antiferromagnetic than ferromagnetic (after removing the S layers at the top layer). Therefore, the undulating magnetic domains of the MFM image due to the ferromagnetic state (Fig. 1d) are considerably related to the 1T-3V_S_ defects. More interestingly, these 1T-3V_S_ defects can be obtained on the sliding surfaces^44^ and the large-area CVD trilayer-MoS_2_ film with the plasma treated substrate^45^. However, there are no 1T-phase-like or V_S2_-like defects on both samples. In the former case, 1T-3V_S_ defects are simulated by rotation of the two single hexagonal lattices by a misfit angle of 30°. The calculated density of states (DOSs) of the 1T-3V_S_ defect doped bilayer MoS_2_ show that the bandgap is closed at the top layer while it is open at the bottom layer (Fig. 3e). In order to investigate the bandgap change due to the electron irradiation, surface-sensitive measurements were performed. Figure 4b–d show that the x-ray photoelectron spectroscopy (XPS) spectra of the electron-irradiated sample shift toward the low binding energy side compared to those of the pristine MoS_2_. However, the stoichiometry of the electron-irradiated sample estimated from the respective integrated peak area of the Mo 3d and S 2p XPS core levels (Fig. 4b,c) retains the ratio (1:2) of the pristine MoS_2_. Deconvolution fits^46^ (Fig. 4b,c) elucidate that both Mo 3d (d_5/2_, 229.77 eV) and S 2p peaks (p_3/2_, 162.58 eV) of the pristine MoS_2_ (component C1 of the 2 H phase) are found to consist of two components after electron irradiation. The intensity ratio of C1 to C2 is estimated to be 0.5. The electron irradiation-induced peaks (component C2 of the 1T-3V_S_ phase) are located at lower binding energies of 229.59 eV (Mo 3d_5/2_) and 162.29 eV (S 2p_3/2_), respectively. It is similar to the 1T-phase doped monolayer^47^. The valence-band maximum (VBM) also moves toward the Fermi energy (E_F_) from 0.99 eV to 0.77 eV as indicated by arrows (Fig. 4d). Notably, the influence of the oxygen, which is inevitable during the electron irradiation, is considered to be negligible from the lack of the change at the weak peak of 236.20 eV (Fig. 4b), corresponding to Mo^6+^ oxidation state of Mo.

**Figure 5:**
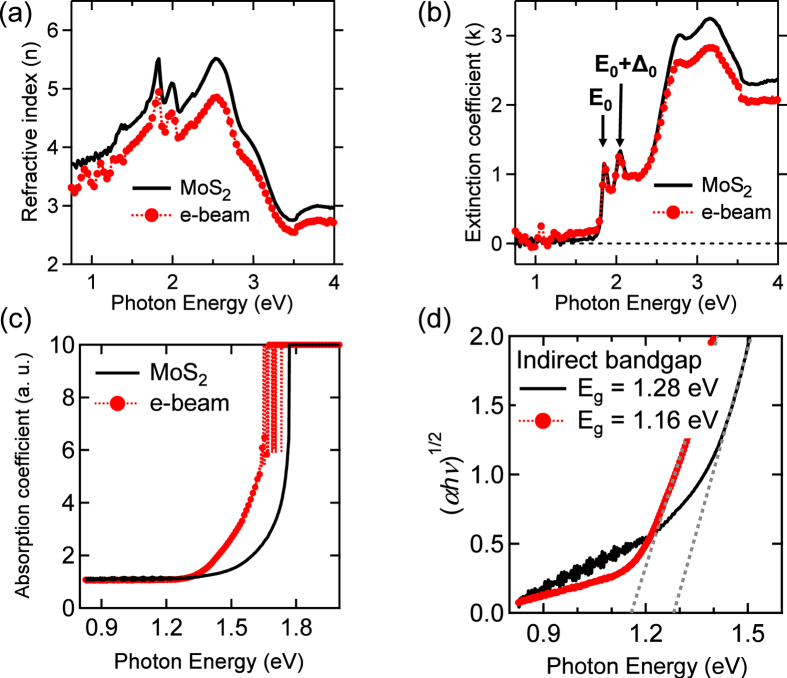
Comparison of (**a**) refractive index *n* and (**b**) extinction coefficient *k* between the single-crystalline MoS_2_ and the electron-sample (300 kGy). (**c**) Optical absorption coefficient shows the dependence of (*ahv*)^1/2^ on the photon energy (*hv*) for two samples. (**d**) Extrapolating the linear part of each curve toward energy axis gives the corresponding indirect bandgap energy (E_g_).

In addition to the shift of XPS spectra toward E_F_, more complementary measurements such as the spectroscopic ellipsometry and optical absorption were measured. Figure 5a,b show the refractive index *n* and extinction coefficient *k* of the spectroscopic ellipsometry, respectively. The sharp feature of Fig. 5b, denoted by E_0_, corresponds to the direct-gap transition at the K point with following by the E_0 _+ Δ_0_ peak, which corresponds to the spin-orbit splitting of the valence band at the same K point^3^. These two features of the direct gap, designated as A and B excitons by PL measurements^2^,^3^, are not responsive to the electron irradiation, while the indirect bandgap shows the oscillating features below 1.5 eV after electron irradiation. The optical absorption results (Fig. 5c) confirm the reduction of the bandgap energy (E_g_) by using the relation: α = A/*hv*(E − E_g_)^n^, where A is the constant, *hν* is the incident photon energy, and the exponent *n* depends on the kind of optical transition^48^. The electron irradiation leads to decrease the indirect E_g_ of the pristine MoS_2_ by approximately 0.12 eV (Fig. 5d), while the change of direct E_g_ is insensitive to electron irradiation as revealed in the spectroscopic ellipsometry. The bandgap reduction is promising in vanishing the Schottky barrier to attaining spintronics device^49^.

## Conclusions

The electron irradiation with the electron dose of 300 kGy (6.70 × 10^14^ electrons/cm^2^) and the acceleration energy of 0.7 MeV creates the 1T-phase-like (V_S2_) and 1T-3V_S_ defects on the MoS_2_ surface. These defects reduce the bandgap and improve the transport property. The undulating magnetic domains of the MFM image due to weak ferromagnetic state are considerably related to the 1T-3V_S_ defects. This optimal electron irradiation to improve the magnetic and transport properties at the atomic-layer scale is a key step for the successful integration of 2D TMDs into possible device applications.

## Methods

### Sample preparation

The natural-single crystalline MoS_2_ samples (SPI) were snipped from a large piece and, after a several exfoliation to take the clean surface, irradiated with different exposure times at the electron acceleration energy (ELV-8 linear accelerators) of 0.7 MeV and 2.0 MeV, respectively, in ambient conditions at room temperature. The area of the electron irradiation at the specific point of 400 ± 50 mm was of width 600 ± 20 × length 20 ± 5 mm^2^ with beam diameter of 25~35 mm. The stability of the beam energy and dose was less than ± 5%. The electron dose was checked by the dosimeter films.

### Characterization

The dc magnetic and hysteresis loop measurements (ca. area of 3 × 3 mm^2^ and thickness of ~100 μm) were performed from 2 to 300 K using a SQUID magnetometer (MPMS XL-7). The MFM measurements were performed with non-contact mode AFM (Bruker-Nano N8 Neo) at room temperature. For the MFM measurements, conductive Pt tips with a radius of ~25 nm were used after Co coating. The MFM images were obtained with a distance of 80 nm between the tip and the sample surface. The electrical conductivity, carrier density, and the Hall mobility were measured as a function of temperature from 100 K to 350 K with a fixed magnetic field of 0.5 T using a Hall measurement system with Au contacts (HMS 5000). HRTEM (JEM-2100F) images for the exfoliated samples by sonication in methanol were taken at an energy of electron beam (200 keV)^40^. The electron irradiation-induced defects are supposed to be hardly influenced by the TEM measurements. From the depth profiles obtained by time-of-flight secondary ion mass spectroscopy, the possible (magnetic) impurities such as O, C, H, and Fe, were found to mostly exist at the electron-irradiated surfaces. There was a negligible reduction of S intensity compared to the Mo intensity on the sample of 300 kGy. Micro-Raman spectroscopy was operated with an Ar ion laser at 514.5 nm. The excitation laser beam of an average power less than 2.5 mW was focused onto samples of interest. The XPS measurements were performed with an Al Kα X-ray source in the vacuum of 1 × 10^−10^ torr (ESCALAB 250XI). The energy calibrations were referenced to adventitious carbon at 284.50 eV with eliminating the charging of the sample during analysis. In fitting of Mo 3d and S 2p core-level spectra^46^, the Gaussian width was fixed at the instrumental resolution of 0.65 eV and 0.60 eV, respectively. The values of the spin-orbit splitting and the branching ratios [I (3d_5/2_)/I(3d_3/2_) and I(2p_3/2_)/I(2p_1/2_)] were 3.17 eV and 0.67 for Mo 3d peaks and 1.18 eV and 0.5 for S 2p peaks, respectively. The refractive index *n*, extinction coefficient *k*, and optical absorption spectra were measured by using spectroscopic ellipsometer (UVISEL) and UV-Vis-NIR spectrophotometer (Cary 5000) in the 300–1600 nm wavelength range at room temperature. Optical absorption coefficient were obtained from the transmission mode at room temperature. The thickness of the pristine MoS_2_ and electron-irradiated sample is ca. 20 and 50 *μ*m, respectively.

### Theoretical calculations

First-principles calculations based on density functional theory (DFT) were performed using the Vienna ab initio simulation package (VASP)^50^. For the exchange-correlation potential, the generalized gradient approximation (GGA) was adopted^51^. Wave functions were expanded by a plane-wave basis set with a cut-off energy of 400 eV. The k mesh in the Brillouin zones sampling is 3 × 3 × 1. We account for bilayer MoS_2_, and a large spacing of between two-dimensional unit cells (15 Å) was employed to avoid interlayer interactions (Fig. 3d). To simulate the 1T-3V_S_ doped bilayer MoS_2_ (Fig. 3c,d), we adopted a 6 × 6 supercell. The in-plane lattice parameter of the bilayer MoS_2_ was used to be the experimental bulk value^46^ of 3.160 Å and the atomic positions were fully relaxed. After gliding three S atoms of the topmost layer along the 2 H to 1 T pathway as indicated by the arrows of the (dotted) circle in Fig. 3b, the concentric-circle-type pattern was constructed by removing the inner three S atoms as shown in Fig. 3c. The bond length and the projected distance (*d*_Mo-S_ = 2.41 Å and 1.82 Å) of the 2 H phase in Fig. 3a increases to 3.72 Å and 3.64 Å in Fig. 3b and to 3.60 Å and 2.74 Å in Fig. 3c, respectively. In Fig. 3d, the bond length (2.41 Å) and bond angle (*θ*_S-Mo-S_ = 80.70°) decrease to 2.38 Å and 79.18° with increasing the *d*_Mo-S_ of the 1T-3V_S_ defect to 2.43 Å and 80.9°.

## Additional Information

**How to cite this article**: Han, S. W. *et al*. Electron beam-formed ferromagnetic defects on MoS_2_ surface along 1T phase transition. *Sci. Rep.* **6**, 38730; doi: 10.1038/srep38730 (2016).

**Publisher's note:** Springer Nature remains neutral with regard to jurisdictional claims in published maps and institutional affiliations.
